# Potency Evaluation and Predictive Quality Control System Construction Strategy for Respiratory Syncytial Virus mRNA Vaccines

**DOI:** 10.3390/vaccines14030199

**Published:** 2026-02-24

**Authors:** Su Zhang, Changgui Li, Yaru Quan

**Affiliations:** State Key Laboratory of Drug Regulatory Science (SKLDRS), National Institutes for Food and Drug Control, Beijing 102629, China; zhangsu@nifdc.org.cn (S.Z.);

**Keywords:** respiratory syncytial virus (RSV), mRNA vaccine, potency evaluation, in vitro–in vivo correlation (IVIVC), standardization

## Abstract

The rapid advancement of respiratory syncytial virus (RSV) mRNA vaccines has created an urgent need for robust, standardized, and predictive potency evaluation systems. Currently, this field relies on diverse, non-standardized in vitro methods that lack quantitative correlations with in vivo immune protection. This poses significant challenges for vaccine process optimization, quality control, and regulatory review. This paper systematically analyzes the strengths and limitations of existing in vitro and in vivo assessment strategies, identifying a bottleneck in the current framework due to the absence of quantitative links between in vitro indicators and in vivo outcomes. It proposes that addressing these challenges hinges on establishing predictive in vitro–in vivo correlation (IVIVC). Furthermore, it outlines a feasible pathway for constructing such predictive models through the design of systematic experimental protocols and multivariate statistical analysis. Alignment with Quality by Design (QbD) principles, this strategy aims to transition potency evaluation from empirical exploration to a predictive, standardized framework, ultimately streamlining the lifecycle management of RSV mRNA vaccines.

## 1. Introduction

Respiratory syncytial virus (RSV) is a major global pathogen responsible for lower respiratory tract infections, posing a serious threat to infants, young children, and the elderly [1]. It is classified within the family *Pneumoviridae*, genus *Orthopneumovirus*, and possesses a non-segmented, negative-sense, single-stranded RNA genome. The viral envelope contains three transmembrane surface proteins: the attachment glycoprotein (G), the fusion protein (F), and the small hydrophobic protein (SH). Among these, the F protein is highly conserved across RSV strains and plays an essential role in viral entry [2], making it the primary target for vaccine development.

The formalin-inactivated RSV vaccine developed in the 1960s was initially regarded as a promising solution. However, it failed in clinical trials because it induced a Th2-biased immune response and carried the risk of vaccine-associated enhanced respiratory disease, characterized by eosinophilic infiltration [3]. This safety setback led to a prolonged hiatus in RSV vaccine development. The field achieved a major breakthrough in 2012 when structural analysis revealed that the metastable prefusion F (pre-F) protein—rather than the postfusion F (post-F) protein commonly found in inactivated viruses—serves as the primary target for highly effective neutralizing antibodies [4]. This discovery laid the foundation for modern vaccine design.

Guided by this breakthrough, from 2023 to 2024, several RSV vaccines based on the pre-F protein have been approved. GlaxoSmithKline’s (GSK) Arexvy and Pfizer’s Abrysvo—both recombinant protein vaccines—were indicated for adults aged 60 and older, with Abrysvo also approved for maternal immunization to protect infants [5]. Subsequently, Moderna’s mRNA-1345, based on the mRNA-Lipid nanoparticles (LNP) platform, was approved for older adults [6]. This technological shift heralds a new era for mRNA vaccines in preventing RSV, while simultaneously introducing novel requirements for evaluating the potency of such vaccines.

Potency is a critical quality attribute (CQA) of vaccines [7], used to control batch-to-batch consistency. Within the framework of regulatory science, the concept of vaccine potency spans the entire product lifecycle and serves as a cornerstone for quality control in global regulatory systems. The core requirement of regulatory authorities is that a vaccine must consistently maintain stable and controllable safety, purity, and potency throughout its shelf life [8]. Among these, potency holds a particularly pivotal position, as it acts as a key bridge connecting the manufacturing process to immunoprotective outcomes. Although the World Health Organization (WHO) defines vaccine efficacy primarily based on the observed reduction in disease risk during clinical trials—focusing on protection under ideal conditions [9]—the regulatory perspective places greater emphasis on ensuring that each commercial batch is released based on stable and reliable testing methods. This ensures that the quality profile of commercial batches matches that of the clinical batches proven to be safe and effective, while also monitoring manufacturing consistency. It is through this rigorous control of intrinsic product quality that regulatory agencies can make scientifically grounded decisions during both pre-market review and post-market lot-release.

The current evolution of vaccine potency evaluation systems has failed to keep pace with the rapid advancement of vaccine platforms. The core issue lies in the absence of validated in vitro–in vivo correlation (IVIVC) capable of reliably predicting clinical outcomes. Unlike traditional protein-based vaccines, where the immunogen is pre-synthesized and potency is directly quantifiable via immunochemical assays, mRNA vaccines essentially “use human cells as factories”. This unique mechanism implies that the mRNA vaccine itself is not the final functional antigen. Consequently, during the regulatory review process, specific in vitro methods must be established to validate transfection-translation efficiency, thereby addressing platform-specific complexities. Precisely for this reason, in vitro potency evaluation must go beyond merely quantifying mRNA levels; it must simulate the entire biological process—from cellular entry and translation to proper protein folding and the expression of antigens with correct conformation. This complexity renders traditional physicochemical assays (e.g., mRNA content quantification, purity analysis, and particle size measurement) insufficient for reflecting true potency, necessitating the establishment of IVIVC models that capture these underlying biological mechanisms. For RSV mRNA vaccines, specifically, the challenge is compounded by the metastability of the Fusion protein. Ensuring the expression of the unstable pre-F is critical for eliciting high-potency neutralizing antibodies. Current research reflects a landscape of diverse, exploratory approaches marked by significant gaps in standardization and predictive capability. Specifically: First, while a multitude of in vitro assay methods have been developed, they lack standardization. Inconsistencies in key elements—such as the choice of cell models and detection technologies—hinder reliable cross-study data comparison. Second, traditional in vivo animal studies, often regarded as the “gold standard”, are too costly and low-throughput to support efficient process development. Third, and most fundamentally, there is a critical shortage of predictive IVIVC models capable of bridging in vitro analytical data with in vivo immunogenicity or protective efficacy.

The fundamental solution to these challenges is to establish a standardized evaluation framework that integrates in vitro screening, in vivo validation, and ultimately evolve into a predictive IVIVC model. Such a framework would aim to accurately reflect vaccine quality and performance, serving as a cornerstone that bridges laboratory research with large-scale clinical application and ensures consistent product potency. Therefore, it is necessary to consider overcoming the bottleneck of fragmented methodologies by incorporating in vitro assessments that encompass antigen content, conformation, and functionality. Providing in vivo evaluations of neutralizing antibody titers will anchor the validation of immunogenicity, ultimately achieving the scientific leap from in vitro indicators to in vivo protective efficacy through IVIVC. This will systematically and comprehensively standardize the potency evaluation methods for RSV mRNA vaccines.

**Figure 1:**
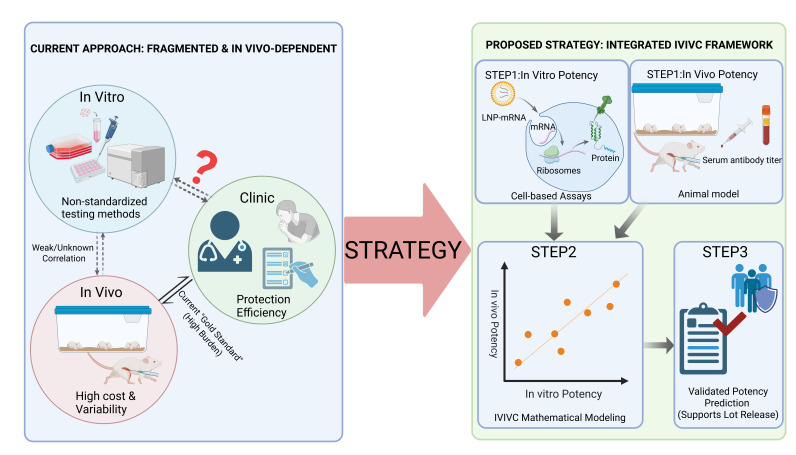
Paradigm shift from a fragmented approach to an integrated IVIVC framework for RSV mRNA vaccine potency evaluation. (**Left**) Current Approach: The existing landscape is fragmented and heavily dependent on in vivo animal studies. While animal models currently serve as the “Gold Standard” for potency assessment supporting lot release, they are associated with high cost, biological variability, and ethical concerns. Current in vitro testing methods are non-standardized, and the correlation between in vitro and in vivo results remains weak or poorly defined. (**Right**) Proposed Strategy: The integrated IVIVC (In Vitro–In Vivo Correlation) framework utilizes both standardized cell-based assays measuring in vitro potency (e.g., antigen expression) and standardized animal models measuring in vivo potency (e.g., serum neutralizing antibody titers) as parallel foundational inputs (STEP 1). These two datasets are essential for establishing the IVIVC Mathematical Model (STEP 2), which quantitatively correlates in vitro measurements with in vivo outcomes. Once established and validated, this model enables Validated Potency Prediction (STEP 3), allowing in vitro data alone to predict in vivo potency for routine quality control purposes, thereby supporting more efficient lot release decisions while reducing reliance on animal testing. In the left panel, the dashed vertical arrows represent the weak or unknown correlation between current in vitro and in vivo methods, while the solid bidirectional arrow indicates the current reliance on animal models as the “Gold Standard” for predicting clinical protection. The red question mark (?) denotes the uncertainty and lack of direct predictive value of non-standardized in vitro methods for clinical efficacy. In the right panel, the red checkmark (√) signifies the successful validation of the potency prediction model, confirming its suitability for supporting routine product lot release decisions.

This paradigm shift is illustrated in Figure 1, which contrasts the current fragmented approach with the proposed integrated IVIVC framework.

## 2. Multidimensional Strategies for In Vitro Potency Evaluation

Guided by Quality by Design (QbD) principles and animal welfare imperatives, the potency evaluation of complex biologics, including mRNA vaccines, is undergoing a paradigm shift. The goal is to replace traditional in vivo assays with robust in vitro markers to ensure product consistency. This transition aims to provide continuous evidence for batch-to-batch consistency and process robustness through robust in vitro indicators. Within this framework, the in vitro potency evaluation of RSV mRNA vaccines requires systematic characterization of three core quality attributes: antigen expression levels, conformation accuracy of the pre-F protein, and its functional immunogenicity. Together, these elements establish a progressive evaluation logic—advancing from fundamental quantification to structural verification, and finally to functional validation—enabling a comprehensive characterization of vaccine quality. This constitutes the essential in vitro indicators for establishing predictive IVIVC.

### 2.1. Precise Quantification of Antigen Expression

The successful expression of the encoded antigen constitutes the fundamental basis for demonstrating the potency of mRNA vaccines. In August 2024, the United States Pharmacopeia (USP) convened its expert committee, in collaboration with industry leaders, to draft an analytical guideline for mRNA vaccine quality. This guideline explicitly recommends cell-based transfection systems for the potency evaluation of mRNA vaccines [10]. Therefore, the primary key to accurately quantifying antigen expression levels lies in selecting an appropriate cell model that strikes a relative balance between transfection efficiency, operability, and physiological and mechanistic relevance. An ideal cell model should exhibit good stability, support efficient transfection and antigen expression, and provide a broad dynamic range for dose–response studies [11].

HEK-293T cells are widely adopted for early-stage mRNA vaccine development, antigen screening, and expression validation, owing to their rapid proliferation and high transfection efficiency [12]. Their utility is demonstrated by their use in the preclinical studies of both Moderna’s mRNA-1273 and Pfizer/BioNTech’s BNT162b2 COVID-19 vaccines [13,14]. While HEK-293T cells are widely adopted for their high transfection efficiency, the selection of cell models should ideally reflect the physiological pharmacokinetics of LNPs. Although mRNA vaccines are typically administered intramuscularly, preclinical biodistribution studies indicate that LNPs can transiently distribute to the liver due to interaction with Apolipoprotein E (ApoE) [15]. Consequently, hepatocyte-derived cell lines such as HepG2 or Huh-7 often yield expression data that effectively mimic robust in vivo translation potential A 2024 study by Nisarg Patel et al. demonstrated that HepG2 cells can generate a complete, sigmoidal dose–response curve for quantifying RSV pre-F antigen expression, with a maximum positive cell rate approaching 90%. This broad dynamic range highlights the cell line’s potential as a preferred model for mRNA vaccine process development and lot release testing [11]. Similarly, Huh-7 cells, another hepatocellular carcinoma-derived line, have shown comparable performance in mRNA transfection and antigen protein expression [16]. As research into the immune activation mechanisms of mRNA vaccines deepens, it has become clear that their protective efficacy depends not solely on the total antigen expression level, but also on which cells—particularly antigen-presenting cells—express the antigen and which (classical or non-classical) presentation pathways are activated. Therefore, selecting appropriate cell lines is crucial for establishing predictive IVIVC models in critical quality attribute studies.

After an appropriate cellular model is established, selecting suitable quantitative methods is essential to obtain reliable data. For this purpose, flow cytometry and enzyme-linked immunosorbent assay (ELISA) are the most widely adopted techniques. Flow cytometry provides quantitative analysis of antigen expression intensity at the single-cell level, whereas ELISA delivers absolute quantification of total pre-F protein from lysed population samples. While these quantitative methods collectively provide indispensable baseline data for potency evaluation, the data they generate cannot elucidate the conformational state or functional potential of the expressed antigen. However, the data derived from these quantitative methods cannot fully elucidate the conformational integrity or functional potential of the expressed antigen. Critically, an antigen that meets quantitative criteria but adopts an incorrect conformation will fail to elicit a robust protective immune response. Therefore, verifying the conformational correctness of successfully expressed antigens is an indispensable subsequent step in the potency evaluation pipeline.

### 2.2. Validation of Antigen Conformation Accuracy

Unlike recombinant protein vaccines, where the immunogen is pre-synthesized, purified, and structurally characterized prior to formulation, mRNA vaccines act as pro-drugs that rely entirely on host cell machinery for antigen translation and folding. As the pre-F form of the RSV F protein is metastable, it readily undergoes spontaneous conversion to the stable post-F conformation during cellular expression. The post-F form exhibits markedly reduced immunogenicity and fails to induce efficient neutralizing antibody production. Consequently, for RSV mRNA vaccines, validating the conformational integrity of the cell-expressed pre-F antigen is central to in vitro potency evaluation. The objective is to confirm whether the vaccine-expressed pre-F protein folds into a functional trimeric structure capable of eliciting potent neutralizing antibodies. Analysis at this stage depends on highly conformation-specific antibodies and a suite of complementary detection techniques.

A 2022 FDA public workshop on advancing nanomedicine highlighted a consensus preference for cell-based protein expression assays in the in vitro potency evaluation of mRNA vaccines. Among available platforms, ELISA and flow cytometry have emerged as the two most widely adopted platforms [17,18], each offering distinct advantages in throughput, informational dimensionality, and potential for standardization.

For the conformational validation of RSV mRNA vaccine antigens, each assay method necessitates the use of specific antibodies coupled with standardized procedures to guarantee both data reliability and cross-platform comparability. Flow cytometry enables direct, in situ analysis of protein conformation on the surface of live cells with high fidelity. Owing to its single-cell resolution and capacity for multiparametric analysis, it serves as the principal method for quantifying cell-surface pre-F antigen expression. The technique yields two critical, simultaneous readouts: the percentage of antigen-positive cells (%Pos) and the mean fluorescence intensity (MFI). This dual-parameter output allows researchers to distinguish between overall transfection efficiency and per-cell antigen expression levels, thereby integrating quantitative analysis with preliminary assessment of conformation. When evaluating mRNA-LNP vaccines encoding the RSV F protein, the Merck team used fluorescence microscopy and image analysis to establish that, in HepG2 cells at 16 h post-transfection, a %Pos > 90% and MFI ≥ 2 × 104 indicates high-efficiency expression [11]. These data establish a valuable reference threshold for interpreting flow cytometry results.

ELISA offers an alternative high-throughput and easily standardized detection approach, with advantages including operational convenience and excellent compatibility with existing platforms in quality control laboratories, making it suitable for rapid screening of large sample volumes [19]. A significant caveat, however, is that the plate-coating and washing steps inherent to ELISA pose a risk of distorting conformational epitopes. Therefore, ELISA is generally not recommended as a standalone method for lot-release testing. Instead, it is effectively deployed for process monitoring or used in conjunction with flow cytometry for cross-validation at critical quality control points—a rigorous strategy to safeguard data integrity.

While conformational validation confirms the structural integrity of the antigen, the ultimate criterion for potency evaluation resides in its biological functionality. Thus, the transition from structural correctness to functional completeness still requires final confirmation through functional activity analysis.

### 2.3. Functional Activity Analysis of Antigens

The final stage of in vitro potency assessment lies in confirming the antigen’s ability to induce functional immunogenicity—that is, its potential to elicit a protective immune response. The competitive ligand-binding assay is a key technique for achieving this objective [20]. The principle of this method involves utilizing the vaccine-expressed pre-F protein to compete with known neutralizing antibodies (e.g., D25, 5C4) for binding to the same type of antibody carrying a detection signal. By quantitatively measuring the degree of inhibition of neutralizing antibody binding, the preservation rate and activity of functional epitopes on the antigen can be assessed [21].

This method generates quantitative readouts such as the half-maximal inhibitory concentration (IC50), which directly reflects the functional immunogenicity of antigens. It serves as a critical functional bridge linking antigen quantity to quality and is one of the highly valuable in vitro indicators for constructing predictive IVIVC models [20]. Within vaccine quality control systems, a functionally validated assay serves as a reliable in vitro screening tool, providing crucial scientific evidence for science- and risk-based quality control strategies.

The in vitro potency evaluation of RSV mRNA vaccines is evolving into a multi-tiered analytical framework, transitioning from simple antigen quantification to a multidimensional platform encompassing antigen expression levels, structural conformation, and functional activity (Table 1). However, for such methods to serve as reliable release criteria, they must comply with relevant guidelines such as ICH Q2(R1) and undergo comprehensive, rigorous validation to ensure accuracy, reliability, and critical comparability. Furthermore, in line with ICH Q6B recommendations, the central challenge of current methodological development is not only to refine individual techniques but, more importantly, to establish quantitative correlations between these diverse in vitro data and the predicted in vivo efficacy of the vaccines [22]. This work enables the forward-looking screening of indicators and accumulation of evidence for building predictive IVIVC models, ultimately providing a scientific rationale for streamlining the release testing paradigm toward a single, robust method.

**Table 1:** Summary of proposed multidimensional in vitro potency evaluation strategies and their relevance to biological performance.

Critical Quality Attribute (CQA)	Recommended In Vitro Assay	Clinical & Biological Relevance
Antigen Expression	Flow Cytometry (cell-based transfection systems)	Reflects transfection efficiency and protein translation levels in relevant tissue cells.
Conformational Integrity	pre-F Specific Binding (e.g., using D25 mAb)	Ensures the antigen maintains the functional prefusion trimer structure required to elicit neutralizing antibodies.
Functional Immunogenicity	Competitive Ligand Binding Assay	Quantifies epitope availability; serves as a direct predictor of in vivo neutralizing antibody titers.

## 3. Paradigms and Challenges in In Vivo Potency Evaluation

While in vitro potency analysis provides critical evidence for quality control, its fundamental value lies in revealing in vivo immunogenicity and ultimate clinical protective efficacy. In vivo potency evaluation remains the primary benchmark for validating the safety and effectiveness of RSV mRNA vaccines and forms the cornerstone of regulatory approval. Its irreplaceable value stems from its capacity to integrate complex in vivo processes—such as biodistribution and immune response dynamics—to provide a holistic evaluation of vaccine-induced immunogenicity and protection. This is the reason that clinical data must be provided before vaccine products can be marketed. For example, Phase III trials of Moderna’s mRNA-1345 vaccine demonstrated an 83.7% reduction in the risk of RSV-associated lower respiratory tract disease [23], offering direct, real-world evidence of its protective benefit. However, in vivo studies are inherently costly, time-consuming, and constrained by interspecies differences, which precludes their routine application in quality control. Consequently, their core utility resides in generating critical “gold-standard” data for establishing predictive IVIVC models and for the screening and validation of preclinical vaccine candidates.

### 3.1. Predictive Value and Inherent Limitations of Animal Models

Animal models provide the essential in vivo response data for IVIVC research. The selection of an appropriate animal model is therefore critical, as it directly determines the model’s ability to predict human responses. An ideal model must achieve a balance among immunological relevance, disease susceptibility, and experimental practicality. Currently, mice, hamsters, and non-human primates (NHPs) constitute the primary model systems [24], each fulfilling a distinct role at various stages of IVIVC development.

Mouse models, particularly the BALB/c *Balb/c* strain, are the primary platform for early-stage screening and large-scale preliminary evaluation owing to their cost efficiency, well-characterized genetics, and the breadth of available experimental reagents [25]. Despite species differences [26], the applicability of BALB/c mice can be enhanced to some extent through the use of humanized transgenic mice or receptor transduction models mediated by viral vectors [27,28]. The data generated sufficiently supports establishing preliminary IVIVCs, making it an efficient platform for verifying positive correlations between in vitro markers and in vivo antibody responses.

The cotton rat (*Sigmodon hispidus*) model exhibits high susceptibility to RSV infection. Following challenge, the respiratory symptoms and pathological changes in this model closely resemble those seen in human infants, rendering it a highly relevant system for evaluating vaccine protective efficacy in challenge studies [29]. In IVIVC research, the challenge protection data and neutralizing antibody titers derived from cotton rats provide the most direct in vivo validation for in vitro functional activity data. Although relatively standardized protocols exist for testing RSV vaccines and antiviral drugs in cotton rat models, their accessibility, cost, and operational complexity pose significant challenges for researchers [30]^,^ thereby limiting the widespread adoption of this model.

NHPs offer the closest approximation to humans in terms of immune system function and overall physiology. Rhesus macaques, African green monkeys, and cynomolgus monkeys are relatively widely used in RSV research [31,32], providing the most physiologically relevant simulation of human immune responses. They serve as a critical bridge for translating preclinical IVIVC models into clinical predictions. Late-stage NHP data in vaccine research carry high authority for confirming whether in vitro assays can predict human immune efficacy. thereby furnishing more reliable evidence to inform clinical trial design. However, the prohibitive costs, complex husbandry and technical requirements, and stringent ethical constraints associated with NHP use preclude their routine application in standard vaccine quality assessment.

In summary, during the establishment of the IVIVC framework, animal models within the IVIVC framework form a tiered validation cascade. Mouse models facilitate the rapid establishment and initial verification of correlations, while cotton rats and NHPs provide rigorous, progressive testing of these correlations in scenarios that are closer to clinical protection. Collectively, this integrated approach furnishes a robust scientific foundation, enabling IVIVC to reliably inform the comprehensive evaluation of RSV mRNA vaccines.

### 3.2. Core Indicators of Functional Humoral Immunity

Despite differences in physiological details among various animal models, they collectively face a fundamental evaluation challenge: translating in vivo immune responses into endpoints clearly correlated with clinical protection. Although RSV vaccines can induce both humoral and cellular immune responses, the lack of standardized methods for assessing cellular immunity and the absence of established quantitative correlations with clinical outcomes currently limit the regulatory application of cellular immune indicators. In contrast, serum neutralizing antibody titer is widely accepted as the biomarker most strongly correlated with protective efficacy [26,33], and is the primary immunogenicity endpoint adopted by major regulatory agencies for RSV vaccine evaluation thereby serving as an ideal bridging endpoint between in vivo and in vitro data.

The clinical relevance of neutralizing antibodies rests on two pillars of robust evidence. First, the practice of passive immunization provides the most direct proof: marketed RSV monoclonal antibodies—from Palivizumab (now largely discontinued) to the next-generation Nirsevimab and Clesrovimab—demonstrate significantly enhanced clinical protection as neutralizing activity increases, with IC50 values decreasing from >100 ng/mL to <5 ng/mL. This reduction correlates with a corresponding rise in protection efficacy, lowering hospitalization rates from 55% to over 80% [34]. This demonstrates the validity of neutralizing antibody titers as a surrogate marker for immune protection, conclusively confirming that neutralizing antibodies alone are sufficient to effectively prevent RSV-related lower respiratory tract disease. Second, for active immunization with vaccines, analysis of Phase III clinical trial data for mRNA-1345 showed that by day 29 post-vaccination, individuals’ RSV-A and RSV-B neutralizing antibody levels increased approximately 8.7-fold and 5.3-fold, respectively. For every 10-fold increase in neutralizing antibody levels, the risk of developing RSV-related disease decreased by approximately 55–59% [35]. Collectively, these findings demonstrate a quantitative, significant positive correlation between neutralizing antibodies and efficacy in preventing symptomatic infection, elevating neutralizing antibody titers to a central position within the field of biomarkers.

In the practical application of IVIVC, standardized neutralization assays—such as the Plaque Reduction Neutralization Test (PRNT) and the Pseudovirus-Based Neutralization Assay (PVNA), and calibrating results to international units per milliliter (IU/mL) using World Health Organization international standards (e.g., NIBSC 16/284) are critical steps to ensure global comparability and accuracy of data. The geometric mean titer (GMT) of neutralizing antibodies, processed through standardization, serves as the key in vivo response variable corresponding to in vitro potency data within the IVIVC mathematical model. This signifies that the ultimate goal of establishing correlations through IVIVC is to enable in vitro indicators to predict clinically validated immune responses representing protective efficacy.

### 3.3. In Vivo Validation of Protective Efficacy Correlation

Challenge studies, which involve inoculating immunized animals with RSV, directly evaluate a vaccine’s ability to reduce infection and mitigate disease severity, thereby providing the most direct evidence of preclinical efficacy. The core evaluation endpoints include viral load in lung tissue and nasal lavage fluid, as well as pulmonary pathology scores. Together, these objective quantitative data determine whether an IVIVC model based on neutralizing antibody titers can accurately predict true biological protection and differentiate the potency levels among vaccine candidates. For example, in the development of the DS-Cav1 pre-F protein vaccine and promising mRNA vaccine candidates, challenge study data provided critical support for the decision to advance into clinical trials [36,37] and laid the foundation for correlating protection outcomes with neutralizing antibody titers.

The aforementioned in vivo protection efficacy and immunogenicity testing collectively establish the cornerstone role of animal models in vaccine development. However, while providing critical evidence, the inherent complexity, high cost, and low throughput of such experiments also constitute disadvantages for routine quality control. This limitation further supports the development of robust IVIVC to correlate such in vivo endpoints with efficient, controllable in vitro assays. Only by establishing a complete chain from in vitro indicators to immune responses and ultimately functional protection can IVIVC evolve from a statistical model into a reliable predictive tool that withstands biological validation. This would enable the construction of a standardized assessment paradigm capable of simultaneously meeting scientific validation and regulatory approval requirements.

## 4. The Necessity of Constructing Predictive IVIVC

The current fragmented evaluation model for assessing RSV mRNA vaccine potency, which involves interpreting in vitro and in vivo data in isolation, is not only inefficient but also faces profound challenges to its scientific foundation. This over-reliance on terminal in vivo studies fundamentally conflicts with the QbD principles endorsed by modern regulatory science, failing to meet the evolving demands of the biopharmaceutical industry.

### 4.1. Limitations of In Vivo Assays for Routine Lot Release

Animal models remain the cornerstone for validating IVIVC during the model establishment phase. However, in the quality control stage, employing them to assess batch-to-batch consistency of in vivo potency for vaccine lot release presents limitations. The current potency evaluation model exhibits deficiencies across three primary dimensions: First, at the scientific level, in vivo experiments suffer from inherent biological variability that often compromises assay resolution. Variations in the genetic backgrounds and physiological states of experimental animals result in high data heterogeneity. For mRNA vaccines, this “biological noise” can mask subtle but critical fluctuations in manufacturing quality—such as minor deviations in LNP encapsulation efficiency or mRNA integrity—thereby failing to provide the discriminative power needed for precise quality control [38]. Second, at the regulatory and efficiency level, reliance on such highly variable methods for lot release not only increases regulatory uncertainty but also undermines supply chain resilience due to the protracted timelines and high costs involved. Third, at the ethical and resource level, the extensive use of animals raises ethical controversies, conflicts with the 3Rs (Replacement, Reduction, Refinement) principle. Regulatory frameworks, such as Directive 2010/63/EU, explicitly mandate that in vivo experimentation must be restricted to scenarios where no scientifically satisfactory non-animal method is available [39]. Consequently, maintaining a quality control strategy dependent on routine animal sacrifice is becoming increasingly unsustainable, as it faces heightened scrutiny and risks non-compliance with these rigorous global standards.

### 4.2. Clinical Significance of IVIVC-Predicted Endpoints

The ultimate goal of establishing IVIVC is to create a standardized framework capable of scientifically predicting a vaccine’s clinical protective efficacy. This process fundamentally involves linking in vitro test indicators to clinical outcomes through a validated mathematical model. To fully appreciate this goal, it is essential to revisit the fundamental purpose of vaccine potency evaluation: it does not directly measure clinical protective efficacy, but rather verifies the robustness of the production process and ensures batch-to-batch consistency. This ensures that every lot released to the market possesses biological activity equivalent to that of the clinically proven batches.

Within the “consistency” paradigm, IVIVC plays an irreplaceable role. As noted, serum neutralizing antibody titer has been established as the key immunological surrogate endpoint for RSV vaccines, with its correlation to clinical protection validated. Consequently, a successfully established IVIVC creates a quantitative link between in vitro potency metrics and in vivo neutralizing antibody levels. This completes a critical logical transition: it demonstrates that determinations of a vaccine’s “consistency” based on in vitro data can effectively translate into reliable predictions of its “consistency in clinical protective potential.”

This conceptual shift fundamentally transforms quality control philosophy. It elevates in vitro testing from a tool monitoring “quality consistency” of product attributes to a predictive instrument capable of forecasting its “clinical efficacy consistency.” Modern quality control is predicated on the principle that products manufactured consistently under a validated, well-controlled system are assured of quality if they are systematically demonstrated to be highly consistent with clinically proven batches [40] IVIVC represents the most scientific and direct means of implementing this principle. It furnishes provides a common scientific language and foundation of trust for enterprises to achieve science- and risk-based lean quality control, as well as for regulatory authorities to conduct efficient and reliable review.

## 5. Core Bottlenecks in Advancing Standardized Effectiveness Evaluation

While a preliminary consensus on the importance and necessity of establishing predictive IVIVC has been reached within the field, its practical implementation continues to face significant challenges. These challenges primarily stem from methodological fragmentation, inherent limitations of in vivo experiments, and inconsistencies in predictive models. To transition from the current exploration of diverse methodologies to a standardized, predictive quality control system in the future, three core challenges must be systematically addressed.

### 5.1. Methodological Heterogeneity Hinders Data Integration

The most readily apparent challenge in the field is methodological fragmentation. Researchers frequently employ disparate approaches in selecting cell models, detection techniques, and even data reporting formats, in the absence of unified standards. This proliferation of non-standardized methods impedes effective cross-comparison and integration of data generated across different platforms and laboratories. For instance, flow cytometry data derived from HEK-293T cells cannot be directly correlated with competitive inhibition results obtained from HepG2 cells. As a result, process transferability, bridging studies for vaccine candidates, and regulatory assessments lack consistent and reliable benchmarks, leading to substantial R&D resource wastage and decision-making uncertainty. Therefore, harmonizing at least a limited set of validated core assay platforms through industry-wide collaboration represents the essential first step toward standardization.

### 5.2. Hurdles in Generating Standardized In Vivo Data for IVIVC Development

Establishing a predictive IVIVC requires not only robust in vitro assays but also a reliable and consistent in vivo dataset to serve as the correlation anchor. However, generating such standardized immunogenicity data faces significant hurdles.

First, the design and execution of in vivo studies for correlation purposes lack harmonization. Critical parameters—such as the choice of animal model, immunization regimen, timing of sample collection, and the specific immunological endpoints measured—vary significantly across studies. This methodological heterogeneity directly compromises the comparability and pooling of data necessary for robust model building. Second, even within a standardized protocol, the intrinsic biological variability of animal models introduces substantial noise, making it difficult to discern subtle but critical effects stemming from vaccine quality attributes.

### 5.3. Challenges in Building and Validating Predictive IVIVC Models

The current challenge in establishing IVIVC stems from an outdated scientific premise. Conventional logic often demands that new in vitro methods establish perfect linear IVIVC with in vivo methods. However, given the inherent and often high variability of the in vivo assays themselves, forcing a strong correlation between a precise in vitro assay and an imprecise in vivo standard is scientifically flawed and statistically indefensible. This rigid “substitution” mindset inherently constrains methodological innovation.

The most fundamental and profound challenge is the widespread lack of predictive IVIVC models. Current research often stops at merely listing in vitro and in vivo data side by side, rather than building a robust, validated mathematical model that quantitatively links rapid in vitro indicators to critical, time-consuming in vivo immune outcomes. In the absence of such predictive models, in vitro data forfeits its prospective value. Consequently, all critical process decisions and lot-release criteria must still revert to in vivo testing. This not only drastically prolongs vaccine development and regulatory timelines but also substantially amplifies project uncertainty and the risk of failure. Therefore, the systematic development and validation of IVIVC constitute the essential pathway to overcome the current evaluation bottleneck and achieve the crucial transition from fragmented methods to a standardized, predictive paradigm.

## 6. Establishing IVIVC for RSV mRNA Vaccines: Precedents and Pathways

### 6.1. Lessons from Established Precedents

Establishing a predictive IVIVC demands a rigorous, reproducible, and systematic research strategy. Valuable insights can be drawn from successful precedents in both human and veterinary vaccine sectors, where in vitro methods have been validated as reliable surrogates for in vivo potency testing.

For human vaccines, the recombinant HPV vaccine provides an instructive model. The IVIVC for HPV vaccines is built upon the in vitro quantification of Virus-Like Particle (VLP) epitope integrity using immunoassays with conformation-specific monoclonal antibodies. Studies have demonstrated that this in vitro antigenicity measurement correlates robustly with in vivo immunogenicity (neutralizing antibody titers in mice), establishing a quantitative relationship that enables regulatory acceptance of in vitro potency assays for routine batch release [41,42]. The success of this approach is predicated on the structural stability of HPV VLPs, which allows for straightforward in vitro characterization of the critical quality attributes that determine immunogenic potency.

In the veterinary sector, the Foot-and-Mouth Disease (FMD) vaccine exemplifies this transition. For FMD vaccines, the 146S antigen content—quantified by sucrose density gradient centrifugation or ELISA—serves as the primary in vitro potency indicator [43]. Regulatory guidelines from the OIE and European Pharmacopoeia recognize the established correlation between 146S antigen payload and in vivo protective efficacy in cattle, thereby permitting the substitution of animal challenge studies with in vitro antigen quantification for routine batch release [44]. This precedent directly embodies the 3Rs principle, demonstrating that robust in vitro analytics can effectively replace in vivo outcomes when a clear, validated correlation is established.

These precedents share a common foundation for success: the target antigens—HPV VLPs and FMD 146S particles—are structurally stable, allowing in vitro assays to reliably capture the critical quality attributes that determine in vivo potency. This structural stability enables a straightforward, often single-parameter correlation between in vitro measurements and in vivo outcomes.

### 6.2. A Tailored IVIVC Pathway for RSV mRNA Vaccines

However, mRNA vaccines introduce unique complexities that distinguish them from these established precedents. Unlike purified protein vaccines (e.g., HPV VLPs) or inactivated pathogens (e.g., FMD vaccines), mRNA vaccines rely on host–cell translation to produce the antigen in situ. This introduces additional variables—including LNP delivery efficiency, intracellular mRNA translation, and the conformational stability of the expressed protein—that are absent in traditional vaccine platforms. For RSV mRNA vaccines specifically, the metastable nature of the pre-F conformation presents a particular challenge, as the antigen must maintain its prefusion state to elicit potent neutralizing antibodies. Therefore, the construction of an IVIVC for RSV mRNA vaccines requires a tailored pathway, which we propose to unfold in three sequential phases.

The first phase focuses on systematically identifying in vitro parameters that are mechanistically linked to in vivo immunogenicity. While the preceding sections of this review have focused primarily on expressed protein-level attributes—particularly pre-F conformational integrity—as the most direct correlate of immunogenicity, a comprehensive IVIVC framework must also consider upstream factors that influence protein expression and delivery. Accordingly, for RSV mRNA vaccines, these parameters span three hierarchical levels. At the mRNA level, integrity, purity, capping efficiency, and poly(A) tail length collectively determine translational competence. At the LNP level, particle size distribution, encapsulation efficiency, and colloidal stability govern cellular uptake and endosomal escape [45]. At the expressed protein level—the most critical and RSV-specific layer—assays must quantify not only the total pre-F protein expression but also its conformational integrity, specifically the ability to maintain the prefusion state rather than triggering to the post-fusion conformation [46]. This requires the use of conformation-specific monoclonal antibodies (e.g., D25, AM14) that selectively recognize pre-F epitopes [47]. Unlike HPV VLPs or FMD 146S antigens, which are structurally stable, the RSV pre-F protein is inherently metastable, meaning that in vitro assays must be carefully designed to detect subtle conformational changes that could significantly impact in vivo potency—a challenge that is unique to RSV and similar metastable antigens.

Beyond mechanistic relevance, practical considerations must also guide assay selection. The precision, throughput, cost, and potential for standardization within a quality control environment must be thoroughly evaluated. Concurrently, reliable in vivo models (e.g., BALB/c mice) must be standardized to minimize biological variability and serve as the reference for subsequent correlation studies. The objective of this phase is to establish a reproducible and transferable in vitro testing workflow that generates reliable data for model development.

The second phase focuses on developing and validating mathematical models that quantitatively link in vitro indicators to in vivo responses. This involves employing analytical techniques such as multivariate statistical regression or machine learning algorithms to explore these relationships [48], with the goal of deriving robust equations that demonstrate stable predictive performance. Given the multi-factorial nature of mRNA vaccine potency, a single in vitro parameter is unlikely to fully predict in vivo immunogenicity. Instead, we advocate for a multi-parameter integration approach, where several in vitro attributes (e.g., mRNA integrity, LNP encapsulation efficiency, pre-F expression level, and conformational purity) are combined into a composite predictive model [49]. Critically, this phase does not dismiss empirical evaluation. Animal immunogenicity studies remain essential during model development to serve as the “gold standard” for training and calibrating the predictive model. The goal of IVIVC is not to eliminate animal testing entirely, but to reduce its routine use in batch release once a validated correlation is established—thereby embodying the 3Rs principle while maintaining scientific rigor.

The third phase entails the rigorous validation of the established IVIVC and its integration into the product lifecycle. Following model development, the IVIVC must undergo both internal and external validation to confirm its robustness and define its predictive scope [41,50]. Internal validation employs statistical techniques such as cross-validation or bootstrap resampling to assess model stability within the training dataset. External validation tests the model on independent batches not used in model development. A key validation strategy involves creating graded sample libraries by introducing controlled artificial stresses—such as thermal acceleration or freeze–thaw cycles—to generate vaccine samples with defined quality attributes ranging from fully potent to partially degraded [26,41,50]. Parallel in vitro and in vivo testing of these libraries yields directly comparable datasets, and analysis of whether the model’s predictions align with the observed trends in in vivo neutralizing antibody titers can thereby confirm the model’s predictive validity [41,50,51]. Beyond initial validation, lifecycle management underscores that an IVIVC is a dynamic, rather than static, framework. It requires continuous monitoring, periodic re-validation, and iterative updates as production processes are refined, analytical technologies advance, or clinical evidence accumulates. This ensures the model retains its predictive relevance throughout the product’s entire commercial lifecycle.

### 6.3. Toward a Standardized Framework: Challenges and Collaborative Imperatives

While the proposed IVIVC framework offers a promising pathway, it is important to acknowledge that statistical and mathematical models, while powerful, have inherent limitations. Model predictions are constrained by the quality and representativeness of the training data, and extrapolation beyond the validated range may lead to inaccurate predictions. Furthermore, complex biological responses may not be fully captured by in vitro parameters alone, and unexpected failure modes may emerge that were not anticipated during model development. Therefore, even with a validated IVIVC in place, periodic verification against in vivo data remains prudent, particularly when significant process changes occur or when the model is applied to new product variants.

Despite these challenges, the establishment of a robust IVIVC for RSV mRNA vaccines remains a goal worth pursuing. Achieving this will require a concerted effort from industry, academia, and regulatory agencies. Collaborative initiatives to share data, validate the most reliable protocols across laboratories, and harmonize acceptance criteria will be essential to build an efficient, reliable, and standardized potency evaluation system for RSV mRNA vaccines. Once a robust IVIVC is established, its impact will be transformative: in vitro assays can predict in vivo efficacy for routine batch release, drastically reducing dependence on animal testing while enabling high-throughput screening of formulations and critical process parameters during process development [52].

## 7. Conclusions

Potency evaluation constitutes the scientific cornerstone for ensuring the consistent quality, safety, and efficacy of RSV mRNA vaccines. The field is now at a critical inflection point, undergoing a necessary paradigm shift. This review systematically analyzes current strategies, highlighting that the traditional model—which relies predominantly on in vivo experiments as the sole indicator of vaccine potency—no longer meets the efficiency demands of modern development and regulatory pathways.

Future competitiveness will be defined by the establishment of a novel assessment system centered on predictive IVIVC. Our analysis identifies three major constraints to this goal: (1) the heterogeneity of in vitro methodologies, (2) the fundamental limitations of in vivo models for routine quality control, and (3) most critically, the urgent need and practical pathway for building predictive IVIVC models.

The path forward is clear: we must move beyond the simplistic juxtaposition and parallel application of existing methods. Dedicated efforts are needed to build a predictive bridge, through rigorous and systematic IVIVC research, that quantitatively links in vitro indicators to in vivo immunogenicity. This transition represents the essential pathway for evolving from a “descriptive” to a “predictive” quality control paradigm.

Therefore, the current focus of RSV mRNA vaccine potency evaluation should be concentrated on the systematic development and validation of IVIVC. This represents not only an urgent necessity for enhancing vaccine development efficiency and success rates, but also a decisive step towards establishing a benchmark for the evaluation of other infectious disease mRNA vaccines, thereby advancing the entire field toward a more mature and reliable stage of development.

## Data Availability

No new data were created or analyzed in this study. Data sharing is not applicable to this article.

## Abbreviations

The following abbreviations are used in this manuscript:

RSV	respiratory syncytial virus
IVIVC	in vitro–in vivo correlation
QbD	Quality by Design
pre-F	prefusion F
post-F	postfusion F
GSK	GlaxoSmithKline
LNP	Lipid Nanoparticle
CQA	critical quality attribute
WHO	World Health Organization
USP	United States Pharmacopeia
ApoE	Apolipoprotein E
ELISA	enzyme-linked immunosorbent assay
%Pos	the percentage of antigen-positive
MFI	the mean fluorescence intensity
IC_50_	the half-maximal inhibitory concentration
NHPs	non-human primates
PRNT	Plaque Reduction Neutralization Test
PVNA	Pseudovirus-Based Neutralization Assay
GMT	geometric mean titer
3R	Replacement, Reduction, Refinement
VLPs	Virus-Like Particle
FMD	Foot-and-Mouth Disease
